# Galveston Medical Club; Differential Diagnosis, Diphtheria and Croup

**Published:** 1886-05

**Authors:** 


### SOCIETY NOTES.

### GALVESTON MEDICAL CLUB.

On December 28, the Galveston Medical Club met in regular session to discuss "THE DIFFERENTIAL DIAGNOSIS BETWEEN MEMBRANOUS CROUP AND DIPHTHERIA."

Dr. SAMUEL A. TOWSEY opened the remarks opon the subject in the following paper :

*Mr. President and Gentlemen:* Speaking generally, we say diphtheria is a malignant sore throat, attended with membranous formation. Few diseases are more dreaded by physician and patient than diphtheria. This disease has been known as long as the history of man exists, but only the last fifty years has it obtained any great degree of notoriety. The epidemic of Bologne, of 1855, 58, and 59, killed 20,000 persons.

It attacks children more than adults, girls more than boys. It has raged epidemically in the dog days, and in the coldest winters equally destructive of life. Is it contagious? Yes; undoubtedly. We know it to be so, although we are not acquainted with the true *modus operandi* by which the contagion operates; but once in possession of houses, or rooms, it clings with a most persistent tenacity. It has never been known to be carried from one house to another, if the person be not affected with diphtheria. The well-known case of Mons. Valleix, the French surgeon, who was attending a little girl with diphthetic sore throat, who was under energetic treatment, and she recovered. However, one day when Mons. Valleix was inspecting her throat, he received into his throat a small quantity of saliva, driven out of that of the patient by the act of coughing. Next day, a little exudation appeared on one of his tonsils. The other tonsil and adjacent parts become speedily covered with false membrane, and in forty-eight hours Valleix was dead from diphtheria.

Another case of diphtheria; the surgeon opened the windpipe without relieving the breathing; sucked the point of incision just made, because there was an obstruction of blood-clots. In forty-eight hours this surgeon was dead. He died of symptoms identical with those of Mons. Valleix.

Trousseau, the celebrated French physician, relates these cases in evidence of its (diphtheria) being contagious.

Trousseau afterward, being inoculated by puncturing his arm, nostrils, and throat, with a lancet moistened with the membrane, which he has just removed from a diphthetic sore throat, proved unsuccessful.

This same experiment was again repeated by another French physician — with the same negative results. That diptheria is a blood disease, and is ushered in by constitutional symptoms as chilliness, elevation of temperature of 2, 3 or 4 degrees, general drowsiness, etc. The space under the jaw will show hard tender lumps; (enlarged glands,) the throat a dark livid color. Uvula much enlarged and swollen, hangs back; a sensation of chilliness every few minutes occurs; stupid feeling, pain in the back, drowsiness, dryness, burning in throat, and inflammation which now begins to spread, and in a few hours the parts become affected, and is prone to spread over upwards to the back of the nose and downwards to the larynx and wind pipe.

At first the membranes are easily detached, but soon become tough and thicker, and when torn off, comes in shreds leaving a raw, bloody surface underneath the removed shreds. Now comes the first heavy breathing, and this is of a most offensive nature; here we have diphtheria, showing the *essential difference* between *that* and "croup" which is the most terrible and fatal of all diseases to which children are liable, and is characterized by inflammation of the *larynx*, accompanied by a membranous growth; this membrane grows in the wind pipe, makes it narrower and smaller, thereby producing a spasmodic tendency in the larynx, caused by the inflamatory action.

It has been thought by many that diphtheria and croup are the same disease. The only difference between them is that in croup the larynx is alone affected by the false membrane; while in diphtheria the membrane grows on other parts as well; the soft palate, the uvula, pharynx included, *i. e.* if a child has the croup the membranous inflammation is confined to the larynx. This disease does not spread to other members of the family; but in diphtheria the case is far otherwise, and nothing is more common than nearly every member of the family to be attacked in turns one after the other. Croup may be considered as a diphtheria of the windpipe, and is a local disease while diphtheria proper is a general and a blood disease.

The time has now arrived that every medical man should make a special study of these two separate and distinct diseases, as the increased population, and the want of a thorough knowledge of diphtheria, renders their assistance often of little or no good at the very moment that medical aid is most needed.

Dr. GOLDMANN opened the discussion by request: Diphtheritis is a disease entirely different from croup—because, while croup attacks only children, diphtheritis respects no age, and grandfather and grandchild may succumb to it on the same day; that croup, if it terminates favorable, leaves no sequel as a general rule, while it is well known, that after diphtheritis a insidious state of anaemia follows, and many other complications are liable to arise, which threaten destruction |to the patient; in croup there is hardly ever endocarditis or myocarditis, which generally accompany diphtheria; nor is the affection of the kidneys, the lymphatic glands and the nerve centres to be observed; croup is generally limited to the air passages—diphtheritis may invade other mucous membranes, or extend itself from the pharynx to the larynx, the buccal and nasal cavities and further on to the nasal duct and eye; or through the eustachian tube to the ear; in the same way it may invade the vagina and extend to the uterus after parturition, and thence to its annexa.

Where the beginning is local, its progress may be compared to that of pustula maligna; but many cases do not commence by showing local manifestations; but instead of it, exhibit all the signs of a severe disease, before any throat symptoms appear. The anatomical changes in diphtheritis are different from those of croupous or catarrhal inflammation. In catarrh it is an excess of the formation of epithelium, which has not time to organize completely, and is shed as fast as formed; in croup it is plastic lymph, which is exuded, and organizes on the mucous membrane, and finally, by its accumulation, leads to stenosis of the larynx; in diphtheria it is an exudation within the substance of the mucous membrane, which, by its pressure, causes gangrene and loss of substance. Consequently after diphtheritis a cicatrix forms; while after croup, in favorable cases, fatty degeneration of the exudation takes place and then gets eliminated. I can not conceive why some practitioners still hold the two diseases to be identical, and often have asked myself whether I was right in being so positive in my assertions on tbis subject. There is one thing deserving the attention of the profession, namely: in what causal nexus this disease stands toward scarlet fever? because some of the severest cases of diphtheria in my practice came in the wake of scarlatina. A remarkable parallel is observed sometimes in measles being followed by nona of which I saw several cases in Mexico. This fact points in the direction of a certain relationship existing between scarlatina and diphtheritis, principally if we take into consideration the glandular affection and that of the kidneys, which are existing in both. The operation of tracheotomy has saved many lives of children, affected by croup; while in diphtheria, the most common occurence is a diphtheritic inflammation of the flesh wound, extensive destruction of tissue, and after all, "exitus lethalis."

Dr. Rogers said that his views harmonized with what had been said, and that the diagnosis of diphtheria was often erroneously made. The manner in which the two diseases are ushered in shows a marked difference, and he was inclined to consider diphtheria a form of internal erysipelas.

Dr. West agreed with what had been said by the foregoing gentleman.

Dr. Kelly, alluding to a paper, published in the Lancet by Sir William Jenner, and to the views of Dr. Jacobi of New York, was inclined to regard diphtheria and true croup as one and the same disease.

Dr. Fry said, that he had had no experience with diphtheria, but thought it possible that the color of the membrane was due to the secretion with which it came in contact. He had seen many cases of *true* croup.

Dr. B. A. Pope said:

*Mr. President:* In advocating the idea of the duality of diptheria and membranous croup, I shall confine myself to the clinical symptoms as they are observed in the conjunctiva of the human eye; leaving the rest of the field of discussion to other members.

In diphtheria there may be no membrane at all, the essential feature being an exudation into the conjunctiva, and even into the deeper parts of the adjacent tissues, affecting even the cartilages of the eye lids. In croupous inflammation there is a distinct and separable membrane or nothing, and never infiltration into tissues, no matter how intense the process.

In diphtheria the membrane, if any, is of a bright yellowish gray color, while in coupous inflammation the membrane is of a grayish white.

If there be a membrane in diphtheritis conjunctivæ it cannot be removed except in parts and in shreds; while in coupous inflammation the membrane is removed without difficulty as a whole, and its color, density and elasticity are different, and strongly contrasting.

In diphtheria the vascularity of the conjunctiva is reduced to a minimum and, being least in the most intense cases in coupous inflammation it is in direct proportion to the intensity of the process.

If a case of diphtheritis conjunctivæ be seen at an early stage, when isolated patches of infiltration exist, the parts of the conjunctiva affected are depressed and never swollen. In croupous exudation the swelling is always great in proportion to the intensity of the process.

In diphtheria (of conjunctiva) the tendency to hemorrhage into the conjunctiva and on the surface in the early stages is very great, which is caused by the mechanical obstruction to the return circulation. In a case of croupous membrane, there is but little tendency to spontaneons hemorrhage, it being produced by the rupture of the blood vessels of the highly congested and swollen conjunctiva, caused by the tearing away of the membrane. The tearing away of membrane in diphtheritis conjunctivæ does not produce bleeding to note.

The cornea almost always is attacked in diphtheria with great probability of perforation, and even of sloughing of the whole membrane. The tendency of the cornea to be injured in croupous inflammation depends on the intensity of the process, and the nature of the disease upon which it has been grafted; and does not seem to be increased by the exudation.

The diphtheritic membrane frequently extends from the conjunctiva to the intermarginal parts of the lids, and in many cases excoriations of the oyter skin of the lids may occur, which take the form of diphtheritic ulcers. This never occurs in croupous inflammation, I believe.

The diphtheritic membrane often extends from the conjunctiva to the nose, and then may extend to the throat. This, as far as I know, does not occur in croupous inflammation.

In diphtheria the lids become in all cases much swollen and rigid; and in bad cases they become enormously swollen, and it is impossible to evert them. They are lurid, hot, glistening, exceedingly painful, and remarkably sensitive to the touch. The carsilages, in young subjects may become so infiltrated that, in the early stages, I have seen the inner surface of the lids thrown into folds and pockets. I have never seen a case of croupous inflammation of the lids, no matter how intense, that presented the above described conditions of the lids.

The secretion (conjunctival) in diphtheria, previous to the stage of ulceration and sloughing of tissue, consists of a thin and scanty yellowish serum, mixed with blood and flaky clots of fibrin. In croupous inflammation the secretion is *mucous*, *muco purulent*, or purulent according to the violence of the process, or the nature of the disease complicated by it.

In diphtheritis conjunctivæ, after eight or ten days, the process of elimination commences, and the surface becomes nodular; this is never the case in croupous inflammation. Ulceration and swelling take place; and with these purulence is established; but all these are only a part of the destructive process, which never occurs in croupous inflammation.

In diphtheritis conjunctivæ, after the last mentioned destructive process has taken place, shrinkage occurs, and entropion and trichiasis follow as after cases of trachoma. These do not occur after croupous imflammation.

Where diphtheritis conjunctivæ arises as a complication, the original disease is lost in it; which is not the case in croupous inflammation.

Diphtheritis is mostly a primitive affection; but in my experience, croupous inflammation is a complication; subordinate to the original affection.

We never see diphtheritis conjunctivæ in one eye, and croupous inflammation in the other.

We do not, in an epidemic of diphtheria, see diphtheria conjunctivæ in one member of a family, and croupous inflammation of the eye in another. Diphtheria produces only diphtheria.

The mildest cases of diphtheritis conjunctivæ do not approach in type croupous inflammation, but retain the typical traits. The most developed cases of croupous inflammation do not resemble diphtheritis conjunctivæ. Cases forming a connecting link are wanting. If there is any relation of cause between these diseases, we must admit that diphtheritis is the most intense, and that the croupous form is a milder type of diphtheritis. If this be admitted, and it cannot be doubted, then there should be a shading of the mild type of diphtheritis into the strongest types of croup; but this is absolutely not true. Each retains its own type, no matter what the intensity of the disease. One of the most complete and typical croupous membranes I ever saw, was in the clinic of Arlt of Vienna. In this case the inflammatory symptoms were extremely mild, and there was but little secretion, and the patient seemed but little annoyed by the process. In looking at this case, one who had ever seen a case of diphtheritis conjunctivæ, no matter how mild, would not have thought to connect that disease with it.

The very fact that we speak of severe and mild cases of diphtheritic conjunctivæ, and severe and mild cases of croupous inflammation is to my mind logically conclusive against the idenity of the two diseases, clinically, or as to their cause.

The treatment of the two diseases, more strikingly than anything else, tends to show their difference. In croupuos inflammation, the use of caustics is, as a rule, indicated; while in all cases of diphtheritis conjunctivæ, no matter how mild, all caustic or irritating interference is almost surely fatal.

Jacobi, I believe, explains the *absence* or *presence* of the diphtheritic infiltration, by the *presence* or *absence* of muciperous glands in the mucous tissue affected; but as the upper eye lids are certainly not wanting in these, and as d. conjunctivitis has its typical development in these lids, it seems that some other explanation will have to be sought for.

Dr. Cook wished to be placed as one who considered the two diseases as distinct.

Dr. Fly also believed that the two diseases were distinct, through as to the result he did know that it mattered whether the doctor said the patient had true croup or diphtheria.

In three cases on which he performed tracheotomy, two of them before the operation, he diagnosed diphtheria; and the wound became diphtheritic; in the third he diagnosed croup and the wound did not take on the diphtheric character.

Dr. Singer said:

*Mr. President:* In our large eastern cities, those physicians who believe in the duality of croup and diphtheria, claim that croup is by far less common, and diphtheria much more so, than it was twenty-five or fifty years ago. This looks very much like a compromise on their part; assuredly it means that even to them the differentiation between the two is extremely difficult.

We all know, that we may have very mild cases of diphtheria; so mild in fact, that unless some special symptoms attract our attention, we may fail to recognize the disease. Ordinarily, diphtheria attacks first the upper air passages, those that are exposed to direct ocular examination; but nothing disproves that it may not primarily show itself in the larynx. Now, being given a mild type of diphtheria, say in a young subject, who exposes himself recklessly to sudden changes of temperature, and whose laryngeal passages may, for that reason, be in a state of cartarrhal inflammation, such condition will no doubt invite the diphtheritic manifestation to this spot; the exudation will form there at once, and all the aymptoms of laryngeal stenosis will appear before the constitutional infection is discovered. Under these circumstances the greatest danger is from asphyxia, and deaths occur from this cause within a very short time; yet that the heart has not been failing, does not disprove the existence of diphtheria. No stress need be laid on the range of the temperature or of the pulse in either case, for authors are far from agreeing on these points; nor is the fact of the glandular enlargement in a well marked case of diphtheria very valuable, for the swelling of the lymphatics depends on the amount of tissue involved by the characteristic lesion, and not on the primary infection of the blood: it follows therefore that where the larynx alone is implicated, we must not look for a glandular enlargement so well marked as that consequent upon a diphtheritic inflammation extending from the posterior nares to the bifurcation of the trachea. It is erroneous, to say, that the so-called croup is a disease of childhood only, for cases of it have been reported as having occured in the adult. It is said, that it differs from diphtheria by not being contagious like the latter; yet epidemics of croup have been reported: facts are also given where inoculations of croupous membranes on animals failed to excite a like inflammation at the inoculated spot. Who will affirm however, that a well marked diphtheritic membrane *al-*

*ways* reproduces diphtheria when applied to a healthy mucous surface? Such a presumption would imply that the origin or cause of diphtheria is a purely local one, a point which is far from being settled at the present day. Furthermore all animals, from man down to the lower ones, are not equally susceptible to the same poison; and it has not yet been proven that any virus taken from the human system can be reproduced with certainty in dogs and rabbits. Cases are reported also of physicians and others having acquired diphtheria through the agency of tracheotomy tubes, used in cases of diphtheritic croup, but it is not related whether anybody has ever been foolhardy enough, to suck a tracheotomy tube used in the so-called membranous croup, for the sake merely of demonstrating that no systemic poisoning would follow. Reports simply tell us, that inspiration practiced through a tube used in a well defined case of diphtheria, *may* carry the disease germ into the system of the reckless manipulator of the instrument, but such poisoning is not always the infallible result. The same argument holds good in such instances of tracheotomy, where diphtheritic inflammation extends to the external wound of the neck; but to affirm that such diphtheritic exudate will occur in every case of diphtheritic stenosis operated on, is trespassing on well established records.

In regard to the sequelœ of diphtheria, such as paralysis, to say the least, they are rare. Yet cases of so-called membranous croup have been reported, which were followed by paralysis. If we admit that in laryngeal stenosis nineteen out of twenty die, how seldom will we be able to see a case of paralysis follow a recovery from this affection?

Much has been argued and said in regard to the respective exudations of diphtheria and croup. Ernst Wagner, several years ago, was the first to proclaim the differences between them, but his views have since then been refuted by Rindfleish and others, and to-day the pathological anatomist makes no distinction between them. Under the microscope we find that the same histological elements make up their composition, and at the bedside the same chemical substances are used to dissolve both. Oertel's far-famed micrococcus is said to be the distinctive characteristic of the diphtheritic membrane; but examinations have been made of non-diphtheritic exudations of the air passages, and micrococci and bacteria have been found in small proportions first, but their number increased from day to day as the examinations were conducted at consecutive intervals. Besides, micro-organisms have been found in the human body independent of any diphtheritic process; and these could in no way be differentiated from the micro-organisms of diphtheria. All that we may conclude from the presence of these organisms is that they indicate a certain amount of constitutional infection; for it is by no means established that they are the disease-conveying germs. Great stress has been laid on the fact that the croupous membrane lies on the mucous surface whilst the diphtheritic lies on, and also within the mucous layer. We are told that in diphtheria, occurring along the whole surface of the respiratory passages, the membrane is incorporated within the substance of the mucous tissue in the larynx and in the parts above it, whilst below the larynx, in the trachea and bronchi, it resembles the croupous exudation by being readily detached from the underlying mucous surface. Why a contipuous membrane should be diphthetic on one extremity and croupous on the other, I cannot understand, unless, I suppose, that one form is merely a gradation of the other; or that the croupous membrane may become a diphtheritic one by the further operation of the morbid process developed in the system.

I do not believe that, even if the duality of these forms of laryngeal stenosis were an established fact, would the treatment of either as a manifestation of the same disorder, namely diphtheria, prove to be injurious to the other form, the croupous. Aside of early tracheotomy, the plan which is conceded to offer the best chances for life, is the attempt to dissolve the obstructive membrane by means of some chemical solvent; the same substance is used by those who differentiate between the membranes; hence there is no danger to the patient to be treated for diphtheritic stenosis, even if he should suffer from such a disease as membranous croup, and it will be readily understood that no diphtheritic process will be treated for a croupous one, by a man who does not believe in the existence of membranous croup.

In conclusion, I will say that I have known two cases of fatal laryngeal stenosis to occur in a family. When the first child was attacked, three experienced physicians named the disease croup, because there was no diphtheria prevalent in the neighborhood. When, a few days later, the second child was similarly affected, and died, the former diagnosis was retracted and that of diphtheria substituted, because "croup, of course, is not contagions." The moral of this tale is obvious.

Dr. FISHER said that he had had no clinical experience with membranous croup or diphtheria, but from what he had read, and from the discussion this evening, especially the convincing argument which Dr. Pope used, he was inclined to consider the two diseases essentially different.

Dr. GWYN said:

*Gentlemen:* But few points have been omitted in the present discussion. 1st, The extreme contagiousness of diphtheria and the non-contagiousness of croup are marked. You have no doubt been frequently called in families where you have met with a severe and even fatal case of croup, while the balance of the family escaped; not so with diphtheria; when once it gains foot hold, rarely an individual escapes.

2d. Another essential difference is the state of the temperature. A fall of the thermometer is hailed with delight in croup, while in diphtheria it is witnessed with gloomy forebodings, and the greater the fall, the more serious the prognosis.

3rd, The mode of death in croup is usually by suffocation. In dyphtheria by many modes.

4th, Croup has no sequelæ, while diphtheria has many, and is itself frequently a sequel of other disease.

5th, Croup is an exudate, while diphtheria is a pathological growth.

I have seen a case of pseudo membraneous laryngitis in a woman of 60 odd years, in which the membranous cast was thrown off.

Dr. LANDEGREN said that at first it was very hard to differentiate the two diseases, but later one presented all the characteristic of a local diseases of a sthenic type and the other a constitutional disease with asthenic symptoms. He considered the two diseases essentially different.

Dr. Goldman closed the discussion by stating, that, notwithstanding the high authorities quoted in contra to his opinion, he could not be convinced of the two diseases being identical. The histological difference of the two affections was undeniable, and also their character; besides this the termination was in one, simply stenosis of the larynx, in consequence of œdema of the glottis and the piling up of exuded membrane; while in the other its fatal exit was generally caused by failure of the hearts action, or by anæmia of the brain. In reply to Dr. Singer "why diphtheria was now so very frequent and croup less so," he wished to state that epidemics had come and disappeared, from time to time, in order to reappear again at a later date. Diphtheria had become general in this country since 1857, and after a terrible epidemic (the black tongue) had prevailed among the cattle, and even the deer on the prairies of Texas and Louisiana, to which thousands fell victims. Many practitioners have dated from this epidemic, the appearance and spread of diphtheria. He was glad to see from the excellent observations of Dr. Pope, that his clinical experience of diphtheritis and croupous inflammation of the conjunctiva did fully confirm what he had stated, as to the pathological difference of the two, inasmuch as he had found, that after the former a loss of substance and a cicatrix followed, while after the latter the membrane returned to its pristine state. The importance of the fact, mentioned by Dr. Pope, was evident, that the cornea becomes invaded and destroyed by diphtheritic inflammation, but not by croupous. The cornea is a cellular tissue within which the exudation forms, and by its pressure interrupts the nutrition, which leads to necrosis; while the croupous exudation limits itself to the surface of the mucous membranes.

Dr. C. H. Wilkinson, President of the Club, closed the discussion with the following remarks :

After the remarks, gentlemen, that have been made upon the subject, but little, if any thing whatever, remains for me to add. Like the majority of those, who have expressed themselves, I regard membranous croup and diphtheria as two separate and distinct diseases, and for the following reasons, to-wit :

1. *Pathologically* considered.

The deposit of croup is an *exudation*, and resembles a simple fibrinous deposit; as in fact it really is. That of diphtheria is an *infiltration*, and rather resembles the accumalation of a syphilitic primary lesion.

The deposit of croup is made *vpon* the mucous membrane of the throat, and no where else; while the infiltration of diphtheria occurs either *within* or *under* that membrane.

In the first instance, the exudate can be forcibly removed without laceration of the subjacent membrane; while in the latter case a raw, excoriated, often bleeding surface, will remain after detachment of the diphtheritic membrane.

Croupous deposit is either not at all impregnated, or sparely so, with bacteriæ; while that of diphtheria is always found *loaded* with them, and the septic condition always found in the blood is clearly and solely attributable to them.

It has been stated that living organisms prevail as well in croupous as in diphtheritic deposits. So they do at times; but never to the same extent in the former as in the latter disease; nor can their migration from the surface to the subjacent blood vessels be traced in the first instance, as they so plainly can be observed in diphtheria. It is very questionable if any exudate or discharge from the human body is always absolutely free from micro-organisms.

Aagain. The deposit in croup is *not innoculable* to any degree of danger to the human system; while that of diphtheria has destroyed many lives by its accidental contact with healthy persons, and is notoriously dangerous in this respect. Otto, Webber, Seekmen, Valleux, Blache, Gillette and other lights in the profession lost their lives by the accidental innoculation of themselves with this diphtheritic poison; and could the entire list be obtained, no doubt many lesser lights could be found to join the foregoing. No such charge can be brought against the deposit in croup. Croup is not innoculable, and does not appear upon the cut surfaces of wounds as does diphtheria.

The cause of death in croup is a *mechanical* interference with respiration, due to occlusion of the air passages by the deposit; while the cause of death in diphtheria is almost invariably due to *heart failure* caused by the presence of septic matter in the blood acting upon it. One therefore is a *mechanical*, while the otber is a *blood poison* or zymotic disease.

Diphtheria is accompanied with *albumin-urea* and often followed by *paralysis*, while no such accompaniment and sequence can be charged to croup. Now, the foregoing pathological differences are well defined, and have been very forcibly stated by Virchow, Buhl, Oertel, Narviloff and other German pathologists, without mentioning our own countrymen, and are, I think, irrefutable. But should any doubt remain as to the non-identity of these two diseases, then *clinical* features should convince all skeptics as to their great dissimilarity.

*Clinically* considered, then croup is *sthenic* in its form and nature, while diphtheria is notably *asthenic*, or the very opposite.

The former is sudden in its invasion attacks children almost invariably, and usually occurs at night. Diphtheria, on the other hand, is oftener gradual in its invasion, attacks adults as well as children, and is as liable to appear, at first, in the day time as in the night.

Any one familiar with the two diseases must have noticed the feeble and compressible pulse, the peculiar palor and the lowering temperature of diphtheria, while but little trouble manifested itself in any of the air passages to create alarm. Mark the contrast in the case of croup! The bounding pulse, excited brain, and elevated temperature in this disease; while the peculiar respiration suggests the mechanical processes at work.

Many other points of difference, gentlemen, could be cited in connection with these two most formidable diseases, but enough have been attended to already, in order to prove their non-identity.

I must congratulate you on the interest you have taken in a theme, the discussion of which may be regarded as really the most important consideration that could occupy the attention of our club at present.

Much has been gained in pointing out the destructive features of the diseases; and with a proper appreciation of their respective natures we surely ought to be the better able to detect the one from the other, whenever we meet them, and thereby be the more capacitated to rescue human lives by treating each for what it really is; and avoiding, thereby, the repeated mortification and disappointment of those who, declaring the two diseases to be identical, very often kill a case of diphtheria while treating it for croup.

